# Reliable microRNA profiling in routinely processed formalin-fixed paraffin-embedded breast cancer specimens using fluorescence labelled bead technology

**DOI:** 10.1186/1472-6750-8-90

**Published:** 2008-11-27

**Authors:** Britta Hasemeier, Matthias Christgen, Hans Kreipe, Ulrich Lehmann

**Affiliations:** 1 Institute of Pathology, Medizinische Hochschule Hannover, Hannover, Germany

## Abstract

**Background:**

During the last years the analysis of microRNA expression patterns has led to completely new insights into cancer biology. Furthermore, these patterns are a very promising tool for the development of new diagnostic and prognostic markers. However, most human tumour samples for which long term clinical records are available exist only as formalin-fixed paraffin-embedded specimens. Therefore, the aim of this study was to examine the feasibility of microRNA profiling studies in routinely processed formalin-fixed paraffin-embedded human breast cancer specimens using fluorescence labelled bead technology.

**Results:**

A statistically highly significant correlation (Spearman r: 0.78 – 0.90, p < 0.0001) was observed for the expression of 319 microRNAs in routinely processed FFPE breast cancer specimens and paired fresh frozen tissue samples (n = 5). Results were confirmed in a larger series analyzing a selection of 10 microRNAs reported to be deregulated in breast cancer (n = 12). The expression pattern of 3 microRNAs was independently validated in this cohort using real-time RT-PCR technology.

**Conclusion:**

Comprehensive microRNA expression patterns can be reliably derived from routinely processed FFPE breast cancer specimens using fluorescence labelled bead technology.

## Background

Formalin-Fixed, Paraffin-Embedded (FFPE) tissue samples represent an invaluable source for the study of human disease. Millions of blocks are archived world wide with corresponding well-documented clinical histories and histopathological reports. The potential value of these archives for retrospective molecular studies has been well recognized [1]. However, the feasibility of every new technology for the molecular analysis of archival FFPE material has to be carefully evaluated using corresponding fresh-frozen material from the very same tissue sample.

The analysis of microRNA expression patterns in human tumour specimens promises to provide completely new insights into tumour biology. In addition, it may contribute to the development of new diagnostic or predictive markers [2,3]. But the vast majority of published studies rely on the analysis of fresh-frozen tissue specimens. Therefore, several studies have addressed the question of microRNA expression profiling in FFPE samples. However, the number of routinely processed clinical specimens analyzed is altogether very low [4-9]. In some studies no fresh-frozen and corresponding archival human material is analyzed [6] or only from a single human tissue specimen [4,5]. All these studies made use of PCR- or array-based methodologies

The quantification of microRNA expression levels using LNA probes coupled to fluorescence labelled beads offers several advantages: No amplification step is required which may introduce a potential bias and the hybridization of probes and target sequences takes place in a homogeneous system [10].

So far, no systematic comparison of microRNA profiles obtained from fresh-frozen and corresponding FFPE samples using the fluorescence labelled bead technology is described.

In this study we examined the expression pattern of 319 microRNAs in routinely processed formalin-fixed paraffin-embedded breast cancer specimens and paired fresh-frozen specimens from the very same tumours. For this purpose the fluorescence labelled bead technology from Luminex (Austin, Texas, USA) was employed.

## Results

### Quality control of RNA and recovery rate of microRNA

The integrity and quality of the RNA preparations was analysed using the microcapillary fluid device form Agilent (Agilent 2100 Bioanalyzer). The mean "RNA integrity number" (RIN)[11] for all 12 fresh-frozen breast cancer specimens was 7.65 (+/- 0.93, range: 5.2 – 8.7) representing quite faithfully the range of routinely processed tissue specimens with high but not perfect quality of the RNA. The highly fragmented RNA from the corresponding formalin-fixed paraffin-embedded tissue samples displayed a mean RIN of 2.26 (+/- 0.28, range: 1.6 – 2.5).

The mean fluorescence intensity, the highest fluorescence intensity, and the sum of all fluorescence intensities were not reduced in any of the FFPE samples compared to the corresponding fresh-frozen sample (data not shown). Therefore, no reduction in microRNA recovery due to formalin-fixation was observed.

### Comprehensive microRNA expression profiling in paired fresh-frozen and formalin-fixed specimens

**Figure 1:**
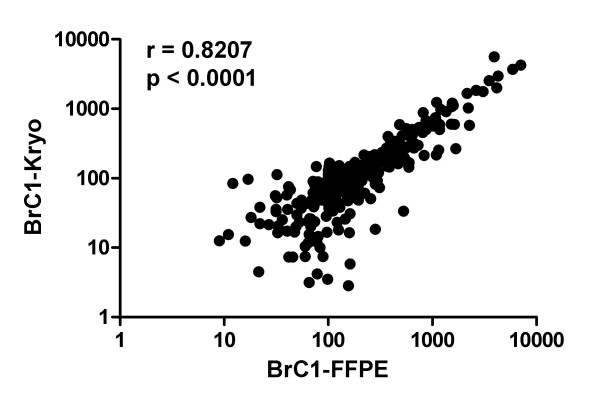
**Comparison of expression levels of 319 microRNAs in paired fresh-frozen and formalin-fixed paraffin-embedded human breast cancer specimens**. The complete data set for BrC1 – BrC5 is shown in Additional files 1 and 2.

The expression level of 319 microRNAs (FlexMir panel version 8 from Luminex) was measured in 5 fresh-frozen breast cancer specimens and corresponding formalin-fixed paraffin-embedded tissue samples from the very same tumour. The measurements showed a very good correlation for all 5 paired samples (Fig. 1 and Additional files 1 and 2). The Spearman rank correlation factors were between 0.6888 and 0.8358 (mean: 0.789) with p-values smaller than 0.0001 for all sample pairs (two-tailed test). Excluding all signals below a background corrected mean fluorescence intensity of less than 100 (arbitrary units of the machine) increased the correlation factors up to 0.8978 (range: 0.7789 – 0.8978, mean: 0.833, see Fig. S1).

### Expression of 10 selected microRNAs in paired fresh-frozen and formalin-fixed specimens

10 individual microRNAs were selected from the literature as most frequently deregulated in primary human breast cancer (as at December 2007, see Table 1, for a recent overview see also [12]).

**Table 1:** microRNAs deregulated in human breast cancer

**miR**	**references**
10b	[16,17]
17-5p	[18]
21	[16,19-21]
27a	[22,23]
125a	[24,19,25]
125b	[16,19,24,25]
145	[16,19]
155	[16,19]
200c	[24,26]
206	[27]

**Figure 2:**
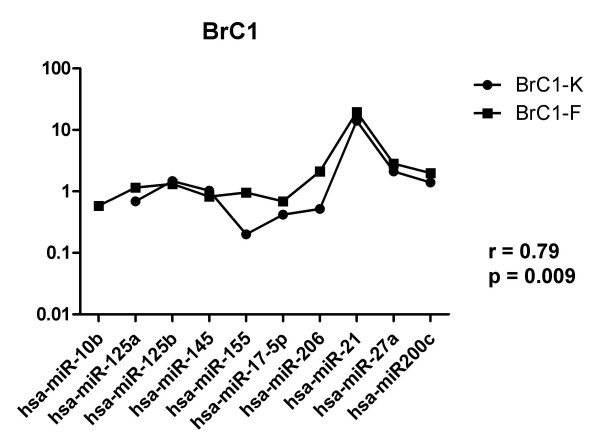
**Comparison of expression levels of 10 selected microRNAs (see Table 1 for selection) in paired fresh-frozen and formalin-fixed paraffin-embedded human breast cancer specimens**. The individual expression levels were normalized to the mean of the four normalization controls included by Luminex (snoRNAs C/D box U13, C/D box U6, C/D box 2, C/D box 6). The complete data set for BrC1 – BrC12 is shown in Additional files 3 and 4.

The expression level of these 10 microRNAs was measured in a series of 12 paired fresh-frozen and FFPE samples (see Fig. 2). Five of these samples were already used for the profiling of 319 microRNAs (see Fig. 1). For eight samples a statistically significant correlation was observed (range of correlation factors: 0.72 to 0.81, mean: 0.77, p < 0.05) whereas for four samples only a trend towards statistical significance (r = 0.41 to 0.62) was observed (The individual p-values for all 12 sample pairs are given in Additional files 3 and 4.). Also, the overall pattern of expression is similar in the four samples without statistical significant correlation. The same microRNAs are up- or down-regulated; only the extent of regulation is different (see Additional files 3 and 4).

### Validation using real-time RT-PCR methodology

The expression level of three microRNAs (miR-10b, miR-145, miR-21) was measured in all samples (12 fresh frozen and 12 FFPE) using the stem-loop primer real-time PCR methodology from ABI (Darmstadt, Germany). Overall, the expression pattern (miR-10b versus miR-145 and miR-10b versus miR-21) obtained with this completely different technique is quite similar: The expression level of miR-145 is only slightly higher than the expression level of miR-10b whereas miR-21 is much stronger expressed than miR-10b (see Table 2).

**Table 2:** Comparison of quantitative real-time PCR results with fluorescence labelled bead technology.

	**miR145/miR10b**	**miR21/miR10b**
qPCR-FFPE	1.3 +/- 0.6	189 +/-137
qPCR-kryo	0.9 +/- 0.4	143 +/- 79
		
beads-FFPE	2.4 +/- 1.3	19 +/- 6.6
beads-kryo	5.5 +/- 3.1	62 +/- 49

Mean expression ratios for three selected microRNAs

However, the extent of overexpression of microRNA 21 relative to miR-10b was approx. four fold higher using real-time PCR (mean: approx. 170-fold) than using the bead-based methodology (mean: approx. 40-fold).

## Discussion

The systematic comparison of paired fresh-frozen and formalin-fixed paraffin-embedded tissue specimens from the very same tumour shows that reliable microRNA expression pattern can be obtained from routinely processed breast cancer specimens using the fluorescence-labelled bead-based technology from Luminex. However, a disadvantage of the current microRNA profiling methodology from Luminex is the requirement of quite large amounts of total RNA (2.5 – 5 μg) which is clearly not available for every interesting case.

The much stronger overexpression of miR-21 as measured by real-time PCR in comparison to the bead-based methodology could be due to slight differences in amplification efficiencies for these two microRNAs. These slight differences in efficiency create a considerable difference in expression level after 25 or 30 cycles due to the exponential nature of the amplification process. Additional sources of differences between the methods compared are the completely different RNA processing (cDNA synthesis versus direct biotin-labelling) and differences in analytical sensitivity (which is higher for real-time PCR). But nevertheless, the overall pattern is the same using both techniques.

Leaving out all signals below 100 arbitrary units improved the correlation quite significantly for three out of five samples, clearly indicating that most of the variability between paired samples are due to fluctuations of the signals of very weekly expressed microRNAs.

Nelson et al. [4] analysed in their pioneering study using their newly developed assay ("RAKE") among other samples a single pair of fresh-frozen and formalin-fixed material from the same tumour (from an anaplastic oligodendroglioma). To the best of our knowledge this represents the very first description of the feasibility of large-scale microRNA profiling in archival samples. A visual inspection of Figure 5 from Nelson et al. gives the impression of quite high concordance between the fresh-frozen and the formalin-fixed sample. However, a formal statistical analysis is not presented and only a single tumour specimen was analyzed.

Li et al. [6] published a comparison of microRNA profiles from snap-frozen cell pellets and formalin-fixed paraffin-embedded cell pellets from an immortalized cell line. The expression level of 160 microRNAs was measured using quantitative real-time PCR and a very good correlation was observed. However, this very well controlled situation does not reflect the routine process with all its uncertainties and variability. Also, the fixation process of a tissue sample is quite different from the fixation of a cell pellet.

Xi et al. [7] studied 254 microRNAs using an LNA-probe array. Analyzing mouse liver tissue the authors found a good correlation between fresh-frozen and formalin-fixed samples (r^2 ^= 0.86 – 0.89). However, no corresponding pairs of routinely processed human tissue samples were analyzed. Only a single archival human colon cancer specimen for which no corresponding fresh-frozen material was available was analyzed.

Wang et al. [8] mention at the end of the results section the analysis of 5 pairs of fresh-frozen and formalin-fixed breast tumours using microarray technology and report correlation coefficients similar to those described in this study (r = 0.443 – 0.927). However, they do not show any primary data and do not provide any data about data validation using a different method.

Lawrie et al. [5] showed in a supplementary figure the analysis of corresponding fresh-frozen and formalin-fixed material from a single lymph node sample using a published microRNA array. Visual inspection of the figure shows a good correlation but no formal statistical analysis is provided by the authors.

The recently published study from Hoefig et al. [9] represents so far the largest direct comparison of microRNA expression patterns obtained from fresh-frozen and corresponding FFPE samples (4 liver and 7 tonsil specimens). The authors used the real-time PCR technology from Applied Biosystems for the analysis of 157 microRNAs. According to the authors, the variance caused by formalin fixation was much smaller than the variance introduced by biological differences (liver versus tonsils). Since the careful data analysis is exclusively based on threshold cycle (C_T_) values and the shift in C_T _values, a direct comparison with the correlation coefficients obtained by us using a different methodology is not possible. However, both technical approaches seem to provide reliable data.

Finally, the evaluation of different microRNA isolation procedures recently published by Doleshal et al. [13], contains also the comparison of microRNA expression levels in paired fresh frozen and formalin-fixed paraffin-embedded specimens using real-time PCR-based methodology, but only for three individual microRNAs.

## Conclusion

The data presented in this study convincingly show that routinely processed human FFPE tissue specimens are suitable for large-scale as well as small-scale microRNA profiling projects using fluorescence labelled bead technology. Therefore, this methodology can now be used for large retrospective studies utilizing stored archival FFPE samples together with the corresponding clinical and histopathological records.

Note added in proof:

During the revision of this manuscript Zhang et al. published the comparison of microRNA expression patterns from fresh-frozen and corresponding FFPE lymph node specimens (n = 7) using the Agilent microarray platform (Agilent, Santa Clara, CA). [14] These authors also found a very good correlation between measurements of matched fresh-frozen and FFPE samples.

## Methods

### Patient samples

12 fresh frozen breast cancer specimens and corresponding formalin-fixed paraffin-embedded tissue samples from the very same tumours were retrieved from the archives of the Institute of Pathology, Medizinische Hochschule Hannover in an anonymous fashion following the guidelines of the local Ethics committee. Tumour cell content was controlled on an HE stained serial section and above 80% for all cases.

### RNA isolation

Total RNA was isolated from fresh-frozen specimens using TRIZOL™ reagent (Invitrogen, Karlsruhe, Germany) following the protocol supplied by the manufacturer. Isolation of total RNA from FFPE specimens was performed as described previously. [15] Following the recommendations from Luminex Inc. no microRNA enrichment was performed.

### RNA quality control

An aliquot of 150 ng of every RNA preparation was checked for integrity in an Agilent 2100 Bioanalyzer (Agilent Technologies, Waldbronn, Germany). Sample preparation and analysis were performed following the manufacturer's instruction.

### microRNA measurement using fluorescence labelled beads

Quantification of microRNAs hybridized to fluorescence labelled beads was performed with a BioPlex 200™ from Biorad (Bio-Rad Laboratories GmbH, München, Germany) using the software Luminex IS™, version 2.3 from Luminex (Austin, Texas, USA). All beads coated with LNA probes complementary to mature microRNAs were purchased from Luminex (Austin, Texas, USA).

Prior to hybridization total RNA is labelled with biotin which is later bound to a streptavidin-phycoerythrin conjugate. For this purpose the FlexmiR™ MicroRNA Labeling Kit from Luminex was used. For the comprehensive profiling of 319 microRNAs (FlexmiR™ human microRNA pool, version 8) 2.5 μg total RNA were labelled, for the analysis of 10 selected microRNAs (FlexmiR™ Select) 0.5 μg were labelled following the protocol provided by the manufacturer. Subsequent washing, hybridization and analysis of samples were performed exactly as described in the protocols provided by Luminex.

The system was calibrated using the xMAP™ calibration control reagents from Luminex following the recommendations of the manufacturer. Every run contained a negative control for background subtraction (water instead of RNA) and a positive control (total human brain RNA from Ambion, provided by Luminex). For data normalization the background value for every individual microRNA bead was subtracted from every corresponding measurement. Subsequently, all corrected mean fluorescence intensities were normalised to pool 1 using the average correction factor for the four normalization controls included in all five pools as recommended by the manufacturer (The 319 different microRNA beads are divided into 5 pools because there are not enough different fluorescence labels available to distinguish more than 70 – 80 beads in a single tube.). These normalised net mean fluorescence intensities were directly compared between paired samples. All control and normalization beads (which are identical in the five different pools) were left out for these comparisons.

### microRNA measurement using real time-RT-PCR technology

For the measurement of the expression levels of selected microRNAs (hsa-mir-10b, -21, -145) the real time-RT-PCR-based detection methodology from ABI (Darmstadt, Germany) was used. Relative expression levels were calculated using the constitutively expressed small RNAs RNU24 and Z-30 as endogenous controls. All primers and probes were purchased from ABI. cDNA synthesis and real-time PCR were performed following the protocols supplied by the manufacturer.

### Statistical analysis

The distribution of the microRNA expression data did not pass the test for normality (α = 0.05) for any specimen. Therefore, Spearman rank correlation factors were calculated (two-tailed). For all calculations the software package GraphPad Prism (version 5.01 for Windows, La Jolla, CA, USA) was used. p-values < 0.05 were considered statistically significant.

## Abbreviations

FFPE: formalin-fixed paraffin-embedded; LNA: locked nucleic acid.

## Authors' contributions

UL and HK conceived the study, BH and UL performed all measurements and evaluated the data, MC and UL performed the statistical analysis and drafted the manuscript, HK selected and evaluated all cases, UL wrote the manuscript with support from HK and MC. All authors read and approved the final manuscript.

## Supplementary Material

Additional file 1 **Comparison of expression levels of 319 microRNAs in 5 paired fresh-frozen and formalin-fixed paraffin-embedded human breast cancer specimens**. The data provided represent the correlation analysis of the microRNA expression levels measured in the paired specimens BrC1 – BrC5 (fresh-frozen versus FFPE). The results for BrC1 are also shown in Fig. 1. Click here for file

Additional file 2 **Comparison of expression levels of 319 microRNAs in 5 paired fresh-frozen and formalin-fixed paraffin-embedded human breast cancer specimens leaving out weak signals**. The data provided represent the correlation analysis of the microRNA expression levels measured in the paired specimens BrC1 – BrC5 (fresh-frozen versus FFPE) leaving out all signals below 100 arbitrary units (see main text for further details). Click here for file

Additional file 3 **Comparison of expression levels of 10 selected microRNAs in BrC1 – BrC6**. The data provided represent the relative expression levels of 10 microRNAs reported to be deregulated in human breast cancer (see Table 1) in the paired specimens BrC1 – BrC6 (fresh-frozen versus FFPE). The results for BrC1 are also shown in Fig. 2. The tumours "BrC1 – BrC5" were also used for the profiling of 319 microRNAs (see Figure 1 and Additional file 1). Click here for file

Additional file 4 **Comparison of expression levels of 10 selected microRNAs in BrC7 – BrC12**. The data provided represent the relative expression levels of 10 microRNAs reported to be deregulated in human breast cancer (see Table 1) in the paired specimens BrC7 – BrC12 (fresh-frozen versus FFPE). Click here for file

## References

[B1] Lewis F, Maughan NJ, Smith V, Hillan K, Quirke P (2001). Unlocking the archive – gene expression in paraffin-embedded tissue. J Pathol.

[B2] Garofalo M, Quintavalle C, Di Leva G, Zanca C, Romano G, Taccioli C, Liu CG, Croce CM, Condorelli G (2008). MicroRNA signatures of TRAIL resistance in human non-small cell lung cancer. Oncogene.

[B3] Lawrie CH (2008). microRNA expression in lymphoid malignancies: new hope for diagnosis and therapy?. J Cell Mol Med.

[B4] Nelson PT, Baldwin DA, Scearce LM, Oberholtzer JC, Tobias JW, Mourelatos Z (2004). Microarray-based, high-throughput gene expression profiling of microRNAs. Nat Methods.

[B5] Lawrie CH, Soneji S, Marafioti T, Cooper CD, Palazzo S, Paterson JC, Cattan H, Enver T, Mager R, Boultwood J (2007). MicroRNA expression distinguishes between germinal center B cell-like and activated B cell-like subtypes of diffuse large B cell lymphoma. Int J Cancer.

[B6] Li J, Smyth P, Flavin R, Cahill S, Denning K, Aherne S, Guenther SM, O'Leary JJ, Sheils O (2007). Comparison of miRNA expression patterns using total RNA extracted from matched samples of formalin-fixed paraffin-embedded (FFPE) cells and snap frozen cells. BMC Biotechnol.

[B7] Xi Y, Nakajima G, Gavin E, Morris CG, Kudo K, Hayashi K, Ju J (2007). Systematic analysis of microRNA expression of RNA extracted from fresh frozen and formalin-fixed paraffin-embedded samples. Rna.

[B8] Wang H, Ach RA, Curry B (2007). Direct and sensitive miRNA profiling from low-input total RNA. Rna.

[B9] Hoefig KP, Thorns C, Roehle A, Kaehler C, Wesche KO, Repsilber D, Branke B, Thiere M, Feller AC, Merz H (2008). Unlocking pathology archives for microRNA-profiling. Anticancer Res.

[B10] Lu J, Getz G, Miska EA, Alvarez-Saavedra E, Lamb J, Peck D, Sweet-Cordero A, Ebert BL, Mak RH, Ferrando AA (2005). MicroRNA expression profiles classify human cancers. Nature.

[B11] Schroeder A, Mueller O, Stocker S, Salowsky R, Leiber M, Gassmann M, Lightfoot S, Menzel W, Granzow M, Ragg T (2006). The RIN: an RNA integrity number for assigning integrity values to RNA measurements. BMC Mol Biol.

[B12] Verghese ET, Hanby AM, Speirs V, Hughes TA (2008). Small is beautiful: microRNAs and breast cancer-where are we now?. J Pathol.

[B13] Doleshal M, Magotra AA, Choudhury B, Cannon BD, Labourier E, Szafranska AE (2008). Evaluation and validation of total RNA extraction methods for microRNA expression analyses in formalin-fixed, paraffin-embedded tissues. J Mol Diagn.

[B14] Zhang X, Chen J, Radcliffe T, Lebrun DP, Tron VA, Feilotter H (2008). An array-based analysis of microRNA expression comparing matched frozen and formalin-fixed paraffin-embedded human tissue samples. J Mol Diagn.

[B15] Bock O, Kreipe H, Lehmann U (2001). One-step extraction of RNA from archival biopsies. Anal Biochem.

[B16] Iorio MV, Ferracin M, Liu CG, Veronese A, Spizzo R, Sabbioni S, Magri E, Pedriali M, Fabbri M, Campiglio M (2005). MicroRNA gene expression deregulation in human breast cancer. Cancer Res.

[B17] Ma L, Teruya-Feldstein J, Weinberg RA (2007). Tumour invasion and metastasis initiated by microRNA-10b in breast cancer. Nature.

[B18] Hossain A, Kuo MT, Saunders GF (2006). Mir-17-5p regulates breast cancer cell proliferation by inhibiting translation of AIB1 mRNA. Mol Cell Biol.

[B19] Volinia S, Calin GA, Liu CG, Ambs S, Cimmino A, Petrocca F, Visone R, Iorio M, Roldo C, Ferracin M (2006). A microRNA expression signature of human solid tumors defines cancer gene targets. Proc Natl Acad Sci USA.

[B20] Si ML, Zhu S, Wu H, Lu Z, Wu F, Mo YY (2006). miR-21-mediated tumor growth. Oncogene.

[B21] Zhu S, Si ML, Wu H, Mo YY (2007). MicroRNA-21 targets the tumor suppressor gene tropomyosin 1 (TPM1). J Biol Chem.

[B22] Tsuchiya Y, Nakajima M, Takagi S, Taniya T, Yokoi T (2006). MicroRNA regulates the expression of human cytochrome P450 1B1. Cancer Res.

[B23] Mertens-Talcott SU, Chintharlapalli S, Li X, Safe S (2007). The oncogenic microRNA-27a targets genes that regulate specificity protein transcription factors and the G2-M checkpoint in MDA-MB-231 breast cancer cells. Cancer Res.

[B24] Scott GK, Mattie MD, Berger CE, Benz SC, Benz CC (2006). Rapid alteration of microRNA levels by histone deacetylase inhibition. Cancer Res.

[B25] Scott GK, Goga A, Bhaumik D, Berger CE, Sullivan CS, Benz CC (2007). Coordinate suppression of ERBB2 and ERBB3 by enforced expression of micro-RNA miR-125a or miR-125b. J Biol Chem.

[B26] Hurteau GJ, Carlson JA, Spivack SD, Brock GJ (2007). Overexpression of the microRNA hsa-miR-200c leads to reduced expression of transcription factor 8 and increased expression of E-cadherin. Cancer Res.

[B27] Adams BD, Furneaux H, White BA (2007). The micro-ribonucleic acid (miRNA) miR-206 targets the human estrogen receptor-alpha (ERalpha) and represses ERalpha messenger RNA and protein expression in breast cancer cell lines. Mol Endocrinol.

