# Supplementary figures and images for: Reliable microRNA profiling in routinely processed formalin-fixed paraffin-embedded breast cancer specimens using fluorescence labelled bead technology
Source: BMC Biotechnol. 2008 Nov 27;8:90. doi: 10.1186/1472-6750-8-90 (PMC2605753; doi:10.1186/1472-6750-8-90)

## Slide 1
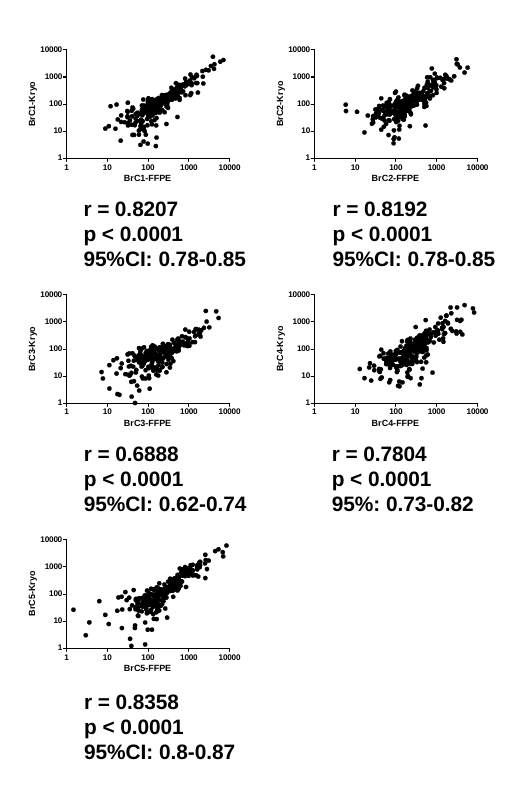

r = 0.8207
p < 0.0001
95%CI: 0.78-0.85
r = 0.8192
p < 0.0001
95%CI: 0.78-0.85
r = 0.7804
p < 0.0001
95%: 0.73-0.82
r = 0.6888
p < 0.0001
95%CI: 0.62-0.74
r = 0.8358
p < 0.0001
95%CI: 0.8-0.87

Supplement: Additional file 1 — Comparison of expression levels of 319 microRNAs in 5 paired fresh-frozen and formalin-fixed paraffin-embedded human breast cancer specimens. The data provided represent the correlation analysis of the microRNA expression levels measured in the paired specimens BrC1 – BrC5 (fresh-frozen versus FFPE). The results for BrC1 are also shown in Fig. 1. [file 1472-6750-8-90-S1.ppt]

## Slide 1
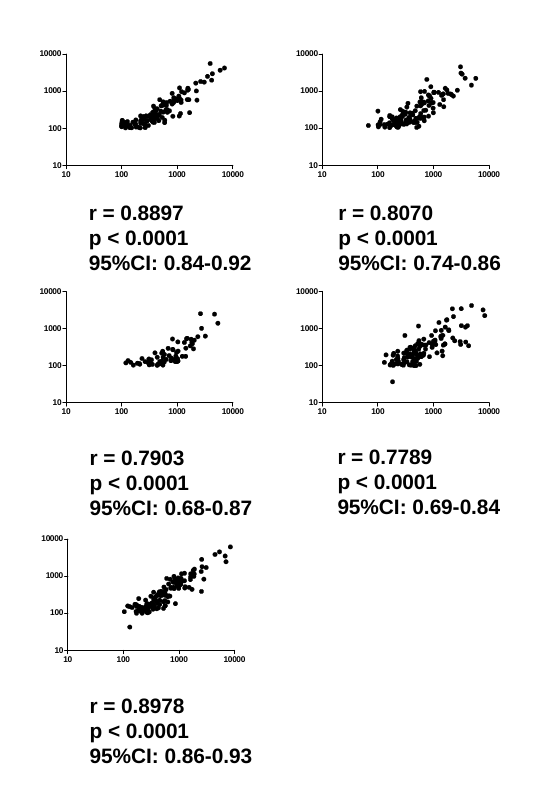

r = 0.8897
p < 0.0001
95%CI: 0.84-0.92
r = 0.8070
p < 0.0001
95%CI: 0.74-0.86
r = 0.7789
p < 0.0001
95%CI: 0.69-0.84
r = 0.7903
p < 0.0001
95%CI: 0.68-0.87
r = 0.8978
p < 0.0001
95%CI: 0.86-0.93

Supplement: Additional file 2 — Comparison of expression levels of 319 microRNAs in 5 paired fresh-frozen and formalin-fixed paraffin-embedded human breast cancer specimens leaving out weak signals. The data provided represent the correlation analysis of the microRNA expression levels measured in the paired specimens BrC1 – BrC5 (fresh-frozen versus FFPE) leaving out all signals below 100 arbitrary units (see main text for further details). [file 1472-6750-8-90-S2.ppt]

## Slide 1
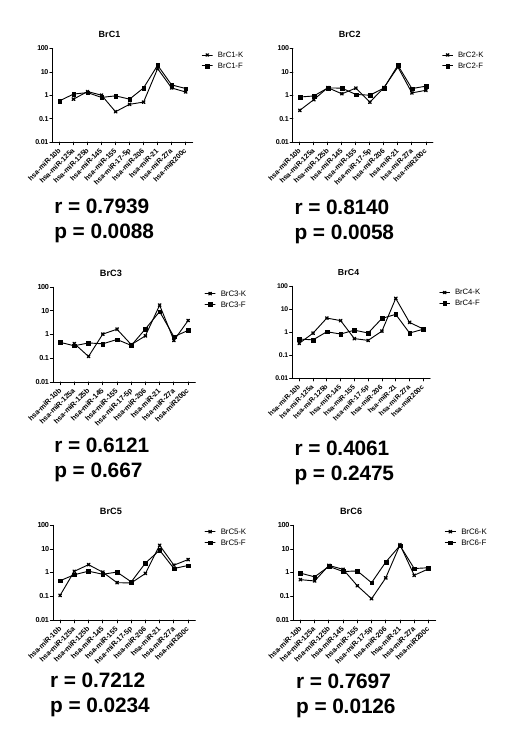

r = 0.7939
p = 0.0088
r = 0.8140
p = 0.0058
r = 0.6121
p = 0.667
r = 0.4061
p = 0.2475
r = 0.7212
p = 0.0234
r = 0.7697
p = 0.0126

Supplement: Additional file 3 — Comparison of expression levels of 10 selected microRNAs in BrC1 – BrC6. The data provided represent the relative expression levels of 10 microRNAs reported to be deregulated in human breast cancer (see Table 1) in the paired specimens BrC1 – BrC6 (fresh-frozen versus FFPE). The results for BrC1 are also shown in Fig. 2. The tumours "BrC1 – BrC5" were also used for the profiling of 319 microRNAs (see Figure 1 and Additional file 1). [file 1472-6750-8-90-S3.ppt]

## Slide 1
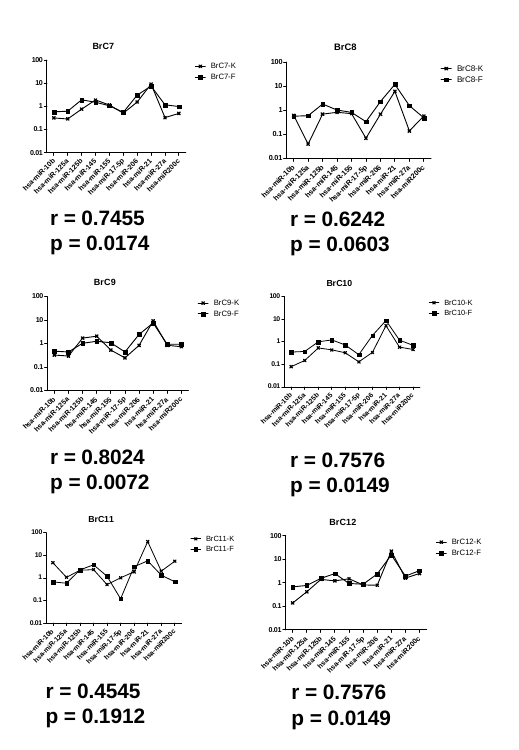

r = 0.7455
p = 0.0174
r = 0.6242
p = 0.0603
r = 0.8024
p = 0.0072
r = 0.7576
p = 0.0149
r = 0.4545
p = 0.1912
r = 0.7576
p = 0.0149

Supplement: Additional file 4 — Comparison of expression levels of 10 selected microRNAs in BrC7 – BrC12. The data provided represent the relative expression levels of 10 microRNAs reported to be deregulated in human breast cancer (see Table 1) in the paired specimens BrC7 – BrC12 (fresh-frozen versus FFPE). [file 1472-6750-8-90-S4.ppt]
